# Understanding the accountability issues of the immunization workforce for the Expanded Program on Immunization (EPI) in Balochistan: An exploratory study

**DOI:** 10.7189/jogh.11.06001

**Published:** 2021-02-11

**Authors:** Zaeema Naveed, Abid Saeed, Aftab Kakar, Faraz Khalid, Nada Alnaji, Gaurav Kumar

**Affiliations:** 1 Department of Epidemiology, University of Nebraska Medical Center, Omaha, Nebraska, USA; 2 Provincial Disease Surveillance & Rapid Response Unit (PDSRU), Provincial Directorate of Health, Quetta, Balochistan, Pakistan; 3 N-STOP (FELTP), Provincial Directorate of Health Quetta, Balochistan, Pakistan; 4 World Health Organization, Geneva, Switzerland; 5 Department of Epidemiology, University of Nebraska Medical Center, Omaha, Nebraska, USA; 6 Department of Health Promotion and Disease Prevention, University of Nebraska Medical Center, Omaha, Nebraska, USA

## Abstract

**Background:**

Among all provinces of Pakistan, immunization coverage is poorest in Balochistan. There is no provincial immunization policy for Balochistan including a lack of human resource management policy. Maladministration and lack of accountability leading to health workforce demotivation and poor performance can be a crucial reason behind an inefficient and ineffective immunization program in Balochistan. The objective of this study was to better understand the accountability issues of EPI workforce at provincial and district level leading to poor program performance and to identify governance strategies for management of inefficiency, demotivation and absenteeism.

**Methods:**

An exploratory qualitative study was carried out to explore issues related to human resource (HR) accountability within immunization program of Balochistan for developing strategies to improve performance of the program. Five districts were selected using purposive sampling based on the comparative poor and good routine immunization coverages and Human Development Index (HDI). Interviews were conducted with EPI Staff and District Health Officers (DHOs) in each district including provincial EPI Staff. A semi-structured and open-ended questionnaire was used. Thematic analysis was used to analyze the qualitative data.

**Results:**

Major barriers to HR accountability included lack of a written HR policy, proper service structure including promotions and benefits and understanding of accurate job description coupled with inadequate HR development budget and activities. Most important demotivating factors were inadequate number of vaccinators, deficient budget with delayed wage and salary disbursements resulting in poor immunization coverage and a lack of appreciation/feedback from senior management for the frontline workers. Key challenge for vaccinators was poor community orientation and mobilization. Although, the participants proposed some solutions based on their perspective, none were elaborate or precise.

**Conclusions:**

Adaptation of National Immunization Policy tailored to the provincial context and proper implementation is much needed. Review of current allocations of vaccinators and need based relocation along with recruitment of new vaccinators with clear job description and terms of reference is desirable. Review of current incentive structure is required. Finally, trust building between community and the vaccination program and social mobilization about the benefits of vaccinations through community influential is vital.

The Expanded Programme on Immunization (EPI) was established in Pakistan as a standalone programme in 1978 [1]. It was executed under the federal Ministry of Health and the national health policy [2] until the country’s 18th amendment in 2010 that devolved legislative and executive functions to the provinces [3]. The amendment, although considered promising with the advent of provincial autonomy, also raised concerns that some provinces might lack the capacity to manage the new responsibilities they were given [4-6]. Today, the local level frontline workforce for routine immunization is comprised of vaccinators who serve in both static facility-based areas and out-of-catchment (outreach) areas [7]. Major providers of immunization services include basic health units (BHUs) and rural health centres (RHCs) [8]. The immunization programme is supported by external resources from donors, such as Gavi, the Vaccine Alliance and UNICEF [9] and local development funds [10].

Although Balochistan is the largest province in terms of land mass in Pakistan, it comprises only six percent of the country’s population. The population is scattered over large, hard-to-reach and security compromised areas. The province has the lowest EPI coverage of all provinces in the country [11]. According to the 2017 Demographic and Health Survey, almost 20% of children aged 12-23 months had all age-appropriate vaccinations [12]. As per the Balochistan’s Comprehensive Multi-Year Plan (cMYP) 2014-2018, there is neither a provincial immunization policy nor a human resource (HR) management policy in place in Balochistan [13]. The cMYP also states that provincial EPI annual plans are not comprehensive enough and there is a Lack of effective communication and coordination among executive district officers (EDOs) and the provincial EPI coordinator [13] (the organogram of EPI Balochistan has been shown in **Figure 1**). There is inadequate number of qualified technical staff for vaccination, surveillance, monitoring, evaluation and cold chain management [14,15]. Furthermore, over-burdened and demotivated vaccinators coupled with delayed fund disbursement and lack of a proper service structure may lead to low performance of the programme.

**Figure 1:**
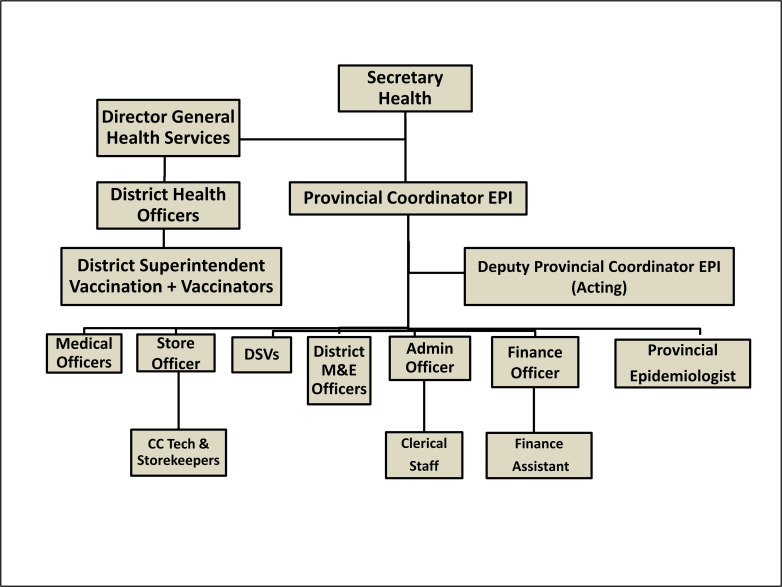
Organogram of Expanded Programme on Immunisation (EPI) Balochistan.

A strong health workforce is a vital component of the World Health Organization’s (WHO’s) health system framework [16]. Demotivation leading to poor performance and absenteeism can be a crucial reason behind an inefficient immunization programme. Apart from lack of funding, weak administration and lack of accountability can be important barriers to the success of immunization programmes [17,18]. In order to develop an effective monitoring, supervision, and accountability system, it is important to investigate the factors that lead to lack of responsibility, especially at the frontline workforce level. This is a crucial knowledge gap at both provincial and district levels for Balochistan’s immunization programme. This study aimed to explore HR accountability challenges hindering immunization service delivery and factors influencing implementation outcomes, as well as identifying potential strategies for management of inefficiency, demotivation and absenteeism.

## METHODS

### Study design and conceptual framework

**Figure 2:**
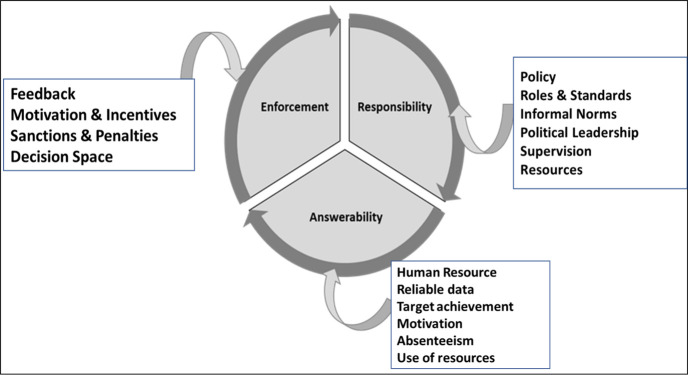
Conceptual framework for the study: Answerability, enforcement, and responsibility.

An exploratory qualitative study was conducted to better understand the perspectives of provincial and district level health officials on HR accountability challenges. The qualitative study design enabled a holistic approach to explore complex realities, latent or evident, constructed by individuals specifically in the context of their everyday interactions [19]. The study was broadly guided by the conceptual framework, which includes the three most frequently described dimensions of accountability in the literature: answerability, enforcement and responsibility (**Figure 2**):

- Answerability determines the condition to inform, elucidate and justify decisions and actions to those affected by these decisions and to those who established the rules [20,21].
- Enforcement regulates the capability to reward good performance or execute sanctions to reprimand bad performance against set standards in an agreement between parties [20,21].
- Responsibility requires that all actors should have clearly defined duties, objectives, and performance standards [20,21].

The elements affecting each of the three dimensions were assigned based on the three types of actors: stakeholders, providers and patients/citizens. These actors play important individual and interlinked roles regarding accountability as described in the health governance framework by Brinkerhoff and Bossert [22]. Stakeholders include policymakers and politicians in health ministries, provincial departments of health and district health management teams. Providers are represented by health workforce operating within primary health care facilities. Patients/citizens are the local community. Combining the dimensions of accountability and the actors involved, the conduct of responsibility advocates changes through actors, including policy makers and managers with formal rules delineating roles, responsibilities, standards to perform against, proper supervision/monitoring and availability of resources. The conduct of answerability advocates changes by providing reliable data, efficient use of supplies and funding, achievement of targets and a decrease in demotivation and absenteeism. Finally, the conduct of enforcement promotes change by motivation and incentives, and sanctions and penalties.

### Study population and sampling

**Figure 3:**
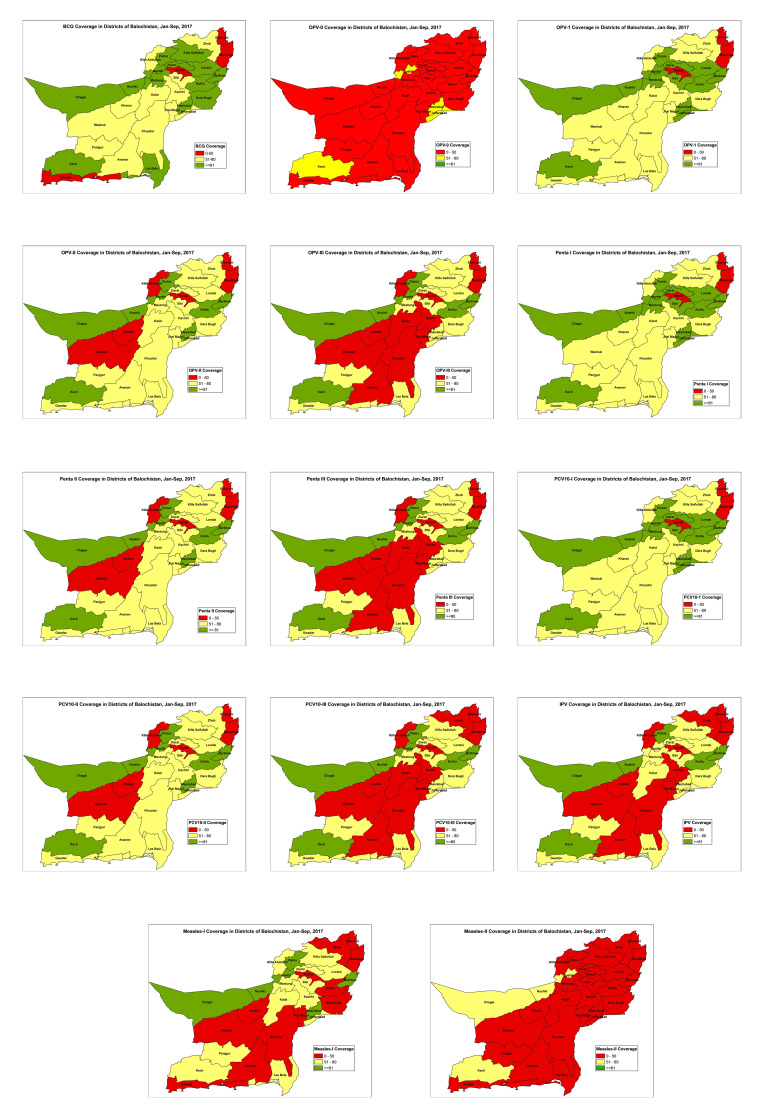
Vaccination coverages for districts of Balochistan, January-September 2017.

Purposive (Maximum variation) sampling was used to select the study areas while random sampling was used to select the study participants from within each district. The five districts, Pishin, Harnai, Jhal Magsi, Killa Abdullah and Killa Saifullah, were chosen based on vaccination coverage as well as on a review of each district’s Human Development Index (HDI). According to EPI administrative data for January and February 2017, Harnai and Jhal Magsi had the poorest coverage for all routine vaccines (**Figure 3**). Additionally, the HDI for these districts were in low (0.29) and medium ranges (0.43) respectively [23]. Killa Abdullah (HDI = 0.24) and Killa Saifullah (HDI = 0.42) are high security risk districts with low immunization coverage and high population movement as they border Afghanistan. Pishin was selected because of its high vaccination coverage (more than 80 per cent for each vaccine) and comparatively high HDI (0.6). This selection enabled the researchers to explore diverse districts to investigate the different issues faced by EPI in Balochistan.

Ten vaccinators were selected randomly from every district except for Harnai and Killa Abdullah, where only five and seven vaccinators were interviewed, respectively, due to saturation of information. Within each district, the total number of vaccinators served as the sampling frame. Every vaccinator was assigned a number and random number generator was used to select a subset. In addition, four district health officers (DHOs), four district superintendents of vaccination/assistant superintendents of vaccination (DSVs/ASVs) and three provincial EPI staff were interviewed. The total sample size was 53, which was enough for result saturation.

### Data collection and analysis

Data were collected at the respective workplaces of the participants. The interviews were conducted in a separate room with only the interviewer and the interviewee present. Interview guides were designed based on the dimension of accountability [22]. The major focus of the questions was to explore the phenomena of responsibility and enforcement, their existence and the effect on answerability. Field guides were were translated into the local language (Urdu) and pretested on two vaccinators per district before administration. Appropriate changes were made to the language of the questions hard to understand. Field guide has been provided as Appendix S1 of the **Online Supplementary Document**.

The questions were semi-structured and open-ended, and the questionnaire was divided into four specific sections. The first section explored the existence and implementation of policy, rules and regulation regarding human resource management and development, along with the availability of material and monetary resources. The second part determined the level of community support and mobilization in relation to the accomplishment of targets by the immunization staff. The next section explored the potential role enforcement could play in enhancing answerability. The last section asked the participants to identify major challenges that hindered their performance and to suggest practical potential solutions.

The interviews were conducted by trained interviewers in local language (Urdu) and were transcribed and translated back into English. The interviews were conducted by the two co-authors of the present study (AK and AS) and their colleagues from the field epidemiology and laboratory training program (FELTP). The interviewers were previously experienced as well as provided further training for the current project. None of the interviewers held a supervisory hierarchical position to the interviewees. Two researchers (ZN and AS) worked separately on translation and developed consensus on key findings. The study was a thematic analysis characterized by coding and categorization of data to explore large amounts of textual information [24,25]. Transcribed documents were entered into MAXQDA12.3.2 (VERBI GmbH Berlin, 2017) and analysed through inductive and deductive approaches. The deductive approach was limited to the broad topics of responsibility, answerability and enforcement that were carried into results as major themes with subthemes that had no predetermined theories for the data supporting them but evolved inductively. The questions were divided under the three domains of responsibility, answerability and enforcement. Open semantic codes were developed within each domain and grouped under categories. Categories with the same contextual information were collapsed and combined into sub-themes. The study was approved by the internal review board of the Health Services Academy.

## RESULTS

The median age of participants was 40 years (mean = 39.5). They had a median education of 12 years (mean = 13.3) and a median work experience of 17 years (mean = 15.9). The results were compiled under four major predetermined themes of responsibility, answerability, enforcement and potential solutions. Each major theme comprised subthemes that emerged through inductive thematic analysis.

### A. Responsibility

#### The non-existence of HR policy

All participants indicated that there is no HR policy document for the EPI. One interviewee stated:

“We do not have anything known as the human resource policy; we don’t even have a provincial health policy let alone the EPI policy.”

#### Documented job description

Majority of participants reported receiving only an appointment letter when they assumed their position, which they called by various terms, such as “order copy,” “job order,” “contract copy” and “job notification.” One vaccinator stated:

“At the time of appointment, we received only one order copy in which it was mentioned that I am appointed as a vaccinator, and my prime job is vaccination. No other description was written on that.”

The participants were of the view that the non-existence of job description makes it difficult for them to understand their responsibilities and thus what is expected of them.

#### Staff training and orientation policies

When asked if they received formal training after being hired, almost half of vaccinators reported receiving it, whereas the others denied receiving it, with some refuting getting any training at all.

“Yes, orientation and training are given at the time of hiring about our job description, roles and responsibilities and vaccination. Then we are attached with senior fellows to train us practically.”

“People might have given orientation and might have been told about stuff, but I don’t know about it, I didn’t get any such thing as training.”

### B. Answerability

#### HR shortage

The problem of a shortage of vaccinators was voiced unanimously. One such response was:

“…the vaccinators are deficient in number, I look after both the static centre, and outreach and my work is affected. It is very evident that due to fewer vaccinators, the staff is overburdened”.

Some vaccinators expressed the need for female vaccinators:

“We have less vaccinators. In Pishin, 12 of our UCs have no vaccinator. It is a widely-scattered district touching the Pak Afghan border; we need more vaccinators and especially female vaccinators who can easily go inside each house and vaccinate each child during outreach activity.”

The shortage demotivates for the vaccinators:

“We are overburdened with the vaccination work due to lack of vaccinators and due to polio campaigns.”

Senior officials had a similar response:

“We have 943 vaccinators available, but our requirement is 1,685. We have demanded it a lot of times from Chief Minister (CM) at each forum and each review meeting.”

About employee turnover, the consensus was nobody resigns and thus the turnover is not responsible for the shortage.

#### Lack of resources

The second major constraint that demotivates and hinders vaccinator performance identified by participants was lack of resources, including material, monetary and logistic resources both for static and outreach activities. Needs frequently mentioned by frontline staff included ice-lined refrigerators (ILRs), bikes and vehicles, petrol, oil and lubricants (POLs), solar panels to keep ILRs running, travelling and daily allowances (TA/DA), and office space.

“We don’t have enough bikes, vehicles, money for their repair and ILRs but the major issues are corruption in funds and resources. Money does come for these things but is not properly utilized.”

Another vaccinator explained:

“We have fewer resources and budget for something like vehicles and POLs for outreach activity. In the past, we used to get 400 rupees per diem, but now we get 200 rupees as TA/DA of outreach. Earlier, we used to get 3 litres of petrol and now 1 litre for our bike for outreach that is a total 13 litres a month. It is not sufficient for covering widely scattered areas of the district for outreach, and this is very demotivating.”

#### Poor community mobilization as a performance constraint

The third major limitation to performance was lack of community mobilization.

“People are not aware of the importance of routine immunization. Doctors are also not cooperative with us, if they refer children to vaccinators in the hospital and educate parents regarding routine immunization it will be good”.

“Social mobilization is very much needed. The community is very rigid; chronic refusals are more in number due to lack of education and awareness.”

#### Availability of district human resource development (HRD) budget

A key complaint that vaccinators made was of the lack of a district HR budget and the absence of a specific person responsible for any such activities. However, some senior staff did mention that partner organizations provide support and budget for the trainings. A few mentioned that district budgets are available, although deficient and varied.

“Yes, I think the budget is there, but we get very less training.”

#### Trainings as a part of HRD

Majority said that trainings do occur but on very irregular basis. However, the rest (n = 14), proclaimed having no training at all. As one vaccinator stated:

“No regular trainings, HRD activities or refreshers are available. These are rare and occasionally conducted when a new vaccine comes or something like that. There is no budget with the district for this.”

### C. Enforcement

#### Salary and benefit structure

When asked about their salary and benefits structure, all participants said that they get a regular monthly salary that is never delayed. However, they did complain about their salary being too low and that there is no defined benefit system including health insurance and paid vacation. About the service structure and promotions, stark variance was observed between higher level and frontline staff. One of the vaccinators said:

“We don’t have a service structure for vaccinators, we get inducted as a vaccinator in scale 5 and retire in the same scale, and there is no promotion system”. In contrast, a senior official stated:

“They are appointed in grade 5 and promote till 16th grade and retire”.

#### Supervision and assistance

With a few exceptions, most interviewees indicated that a district supervision plan is in place, however, some questioned whether the supervisory plan is followed to the fullest.

“This thing is not on the ground, only in papers everything is present, and we usually get monitored and supervised only during the campaigns.”

Third-party monitors were also mentioned about monitoring activities.

“At district level, DHO, DSV, ASV, WHO, UNICEF and monitors from provincial EPI do the supervision and monitoring of all union councils.”

#### Disciplinary measures and accountability procedures

Most front-line workers denied having any specific discipline procedures or protocols and were of the view that DHOs do whatever they consider best. A vaccinator mentioned:

“DHO is the authority, if any complaint comes, he sets up inquiry and asks for explanation but there have never been any terminations. I think there may be a system, but we never saw it being implemented.” However, one senior official mentioned an interesting and possibly effective intervention to avert absenteeism and to monitor performance known as E-Vaccs that Punjab is already using.

“To monitor the performance of vaccinators, EPI Balochistan has taken support from Punjab Institute of Technology Board (PITB), they have provided us a software (E-Vaccs) free for three years. This has created a problem for vaccinators who didn’t perform honestly. Now, 25 vaccinators of Quetta district have *been issued show-cause notice by secretary health and pay has been stopped.”*

#### Performance evaluation

Most respondents said there is no such thing as performance evaluation or refresher training.

“There is no performance evaluation for us. We were told they will start in 2017 but still not started”.

An opposing statement came from another vaccinator from Kila Saifullah.

“Yes, each month EPI review meeting is held with the support of UNICEF for performance evaluation by district EPI coordinator supported by UNICEF.”

#### Lack of appreciation and incentives

The most common sources of demotivation mentioned by the respondents were lack of appreciation and lack of performance-based incentives. Participants discussed the importance of incentives not just in the form of money, but also in terms of promotions, salary bonus, verbal appreciation, prizes and certificates.

“Indeed, it plays an important role, if the staff is working hard and he/she is dutiful, and still he/she is not appreciated and not given any incentive, prize or certificate he /she is badly discouraged and demotivated.”

#### Lack of feedback and remedial actions

Another major source of demotivation identified was lack of feedback and corrective actions based on data and complaints submitted by the vaccinators.

“We always send our reports, issues, and recommendations to higher-ups, but we never get any feedback or action on highlighted issues to date.”

#### Lack of monetary and material resources

Another major demotivator was lack of budget for material resources. As one vaccinator explained:

“We have fewer resources and budget for something like vehicles and POL for outreach activity. In the past, we used to get 400 rupees as periderm, but now we get 200 rupees as TA/DA of outreach. Earlier, we used to get 3 liters petrol and now 1 liter for our bike for outreach that is a total 13 liters a month. It is not sufficient for covering widely scattered areas of the district for outreach, and this is very demotivating.”

### D. Solutions to major challenges

As shown above, the major challenges identified include lack of resources, lack of community mobilization, deficient vaccinators, lack of appreciation, poor monitoring and supervision, difficult geographical access, unclear roles and responsibilities, absenteeism and delayed corrective actions. Participants identified some solutions to these specific challenges. However, most of the solutions were brief and generalized with no specification of time, place or person responsible.

“Female vaccinators are scant in our province. There should be more employment of female vaccinators so that they may also go inside the houses.”

“Religious leaders should be more involved to create awareness about vaccination in the community and government should instruct teachers, and they should tell parents about routine immunization.”

“DHO and DSV should pay more visits to the centres and outreach to supervise and monitor.”

“There should be a separate committee for ensuring accountability in the EPI with new measures.”

## DISCUSSION

Vaccination coverage in Balochistan is significantly lower than other regions in Pakistan, 18.6 per cent vs 65.6 per cent in Punjab, 35.4 per cent in Sindh and 37.1 per cent in Khyber Pakhtunkhwa (KP) [12]. This alarmingly low vaccination coverage requires an urgent plan of action to improve the EPI in this region. HR accountability issues may contribute to the current challenges of the EPI and resulting low coverage.

The current study aimed to explore these HR accountability issues in the context of local settings with key stakeholders working in EPI in Balochistan. The study utilized qualitative methods to investigate the factors associated with the HR accountability in Balochistan and revealed that HR accountability is complicated and is affected by different factors ranging from availability of human and material resources to community mobilization and participation, with everything having a basis in the EPI policy. In addition, the wide variety of factors affects motivation and sense of responsibility of the front-line staff, resulting in poor immunization coverage. The existing literature led us to develop a conceptual framework (**Figure 1**) that encompasses the three major dimensions of accountability that not only govern associations of various human resource issues but also govern the actors fundamental to each dimension.

Perceived challenges for EPI staff and managers can be classified into three levels: staff level, management level and community level. At the staff level, the EPI in Balochistan suffers from a severe shortage of staff and current staff members are overworked and are not able to fulfill the high demand. The shortage becomes even more troublesome when vaccinators are employed in additional polio immunization campaigns. The adverse effect that staff shortages can have on immunization coverage in other settings has been established in the literature [26,27]. As mentioned in the Balochistan’s cMYP and by one of the participants, the district and provincial health departments need to prioritize the issue and accelerate the planned recruitments. Furthermore, a review of the allocation of existing human resources and reallocation if possible, could be another way to address staff shortages. The results did not find staff turn over to be a problem but rather creating positions and hiring.

In addition, most participants complained of low salaries, lack of career opportunities provided by the EPI. The issues of staff shortages and low salaries are very often encountered in developing country settings [28,29]. Although financial incentives to staff should be considered, more innovative non-financial incentives should be utilized by the district government to ensure that the staff members are motivated to deliver sustainable quality services. Evidence from other developing countries in utilizing non-financial incentives shows great promise. The community health workers in Ethiopia have high level of motivation through the use of non-financial incentives such as ongoing mentoring, training, certification, awards (non-financial) and celebrations [30]. Similarly, Kenya also showed that non-financial incentives play an important role concerning increasing motivation of health professionals [31].

At the management level, the lack of clear job description, formal training, supervision and accountability process were all challenges that emerged in the results. The need for a clear job description was identified in the evaluation of other health programmes in Pakistan [32], suggesting that this is not unique to Balochistan Province. A clear job description is vital to assess the achievements against the expectations/responsibilities. A study conducted in Georgia found that provider-based interventions, such as supportive supervision, can have positive effects on immunization programme indicators [33]. Staff training and refreshers were mentioned by many respondents, and dissatisfaction was expressed by most. Both at the federal and district level, the HR policy needs to incorporate a proper and comprehensive training schedule not only about vaccination schedules but also administration, storage, adverse events following immunization (AEFI), record keeping and communication skills.

Although the research questions were not focused on community mobilization, this topic emerged in several interviews. Community mobilization is the process of bringing together all societal and personal influences to raise awareness of and demand for health care, assist in the delivery of resources and services, and cultivate sustainable individual and community involvement [34]. Important barriers for community mobilization that were identified in this study included the lack of community awareness on the importance of vaccinations, and the lack of support and coordination between health providers and EPI services. Further research is needed specifically in context to province of Balochistan that is characterized by unique cultural and political dynamics compared to other parts of the country, to evaluate the most common barriers to community mobilization, and the lack of trust between the community and the vaccination programme. This was out of the scope of the current study. Most of the best practices in the literature are based on community mobilization through education and information dissemination and are found to have significant improvement in vaccination coverage [35-38].

The credibility of the findings was ensured using multiple procedures. The data collectors had no difficulty in establishing prolonged engagement as they work with the health department and understand the culture. Source and analyst triangulation were established. Different cadres were interviewed for information and data was analysed independently by two researchers to look at different aspects of information. Due to time limitations, no negative case analysis or member checks and external audits could be performed. Although the districts were selected purposely to have maximum variation so that it is more generalizable at the provincial level, there may still be unexplored factors specific to other districts.

## CONCLUSION

The findings of this study suggest that the non-existence of a provincial HR policy is seen as a main barrier to programme performance in Balochistan. There, it is recommended that Pakistan’s national immunization policy be adapted by the Balochistan provincial health department including a detailed description of rules, regulations and procedures based on the local context. Development of clear job descriptions and TORs followed by hiring and appointments of new staff members should be initiated as soon as possible preceded by a proper need assessment of resources. This desires a bottom-up approach starting at each district steered by EDOs with stewardship and approval at the provincial level. In addition, findings of this study strongly support the need for modification and proper implementation of a training schedule not only on vaccine administration but also on vaccine management and communication skills. Record keeping is vital to ensuring that staff is accountable for practicing what they learn in training sessions. Another important intervention to boost the motivation and sense of responsibility of the front-line workers is a well-defined incentive and appreciation system. Lastly, it is recommended that a comprehensive community mobilization programme is designed and implemented. In the context of Balochistan, educational sessions, postal or telephonic reminders, and parental incentive schemes (some monetary benefits on initiating and then regularly following up for updating immunization status of the child) may prove to be successful.

## Additional material

Online Supplementary Document
