# Supplementary material for: Understanding the accountability issues of the immunization workforce for the Expanded Program on Immunization (EPI) in Balochistan: An exploratory study
Source: J Glob Health. 2021 Feb 11;11:06001. doi: 10.7189/jogh.11.06001 (PMC7916446; doi:10.7189/jogh.11.06001)
Supplement: Online Supplementary Document [file jogh-11-06001-s001.pdf]

Appendix S1.

IN DEPTH/KEY INFORMANT INTERVIEW GUIDE (Vaccinator)

PROJECT INFORMATION	
Study Title	Understanding the accountability issues of the immunization workforce for the Expanded Program on Immunization (EPI) in Balochistan: An exploratory study
Location	Balochistan [Pishin, Harnai, Jhal Magsi, Killa Abdullah and Killa Saifullah]
Duration	6 months
**INVESTIGATOR INFORMATION**	
**1. Principal Investigator**	
Name:	Dr Aftab Kakar

In-depth/key informant interview guide for policy makers, EPI programme managers and frontline workers at provincial and district level on topic entitled 'the measures/governance strategies that can lead to a strengthened human resource accountability of immunization program in Balochistan to manage demotivation, inefficiency and absenteeism'.

Overall questions to be answered during these interviews are:

- To gain insight about existing rules regarding roles, responsibilities, and standards for service delivery actors and identify the gaps
- To explore political leaders and community owners support for routine immunization having potential impact on human resource motivation and efficiency
- To determine the role of incentives/motivation and penalties/sanctions in improving performance
- Synthesize the current best evidence on effective measures of strengthening internal accountability

INTRODUCTION

Hello! I am XXX and we are doing research on topic entitled, measures/governance strategies that can lead to a strengthened human resource accountability of immunization program in Balochistan to manage demotivation, inefficiency, and absenteeism.

We are conducting this research because we are interested in finding out the keys issued pertaining human resource management and policies and to come up with evidence-based suggestions to have strengthened human resource accountability of Balochistan's immunization program. We expect that the knowledge gained will be used to highlight the factors effecting the performance and immunization services and will ultimately be helpful to device realistic solutions at various levels of the program.

You were chosen to participate because you hold an important role to play at the frontline level and are responsible for actual implementation of EPI activities. Interviews enable us to get more in-depth information about a particular topic. We are very interested to hear your input. If it is okay with you, I will be recording our conversation. The purpose of this is so that I can get all the details,

but at the same time be able to carry on an attentive conservation with you. Only the research team will have access to the tapes, and they will be destroyed as soon as they are transcribed.

The information that you give us is completely confidential. We will not associate your name with anything that you say. If you do not understand a question, please let us know. You may refuse to answer any questions or withdraw from the study at any time.

Do you have any questions?
Yes / No

I would like to start by having you briefly describe your role and responsibilities of sharing information and views.

DEMOGRAPHICS

Name of interviewee:

Position Title:

Age:

Gender:

Education:

Work experience:

Type & Name of facility:

Address of the facility:

Interview conducted at (place):

RESPONSIBILITY

I am now going to ask you some questions about HR management that I would like you to answer to the best of your ability. If you do not know the answer, please feel free to say so.

1. What is your opinion of the human resource policies and practices of Baluchistan's immunization program?
   <u>Prompts and probes:</u>
   a. Does a written policy manual exist about roles and responsibilities, compensation and benefits and is it available to all of you?
   b. Did you receive a written contract with job description and organizational policies?
   c. Is there a proper system to determine salary scales and benefits?
   d. Are formal systems, monitored and used in all hiring, transfer, and promotion decisions?
   e. Is orientation offered to all new employees emphasizing the mission and performance standard expected?
   f. Do formal procedures regarding discipline and termination exist and are they properly followed?
   g. Is there a proper system to collect and store employee data? Are employee records stored and available?

   h. How important, in your opinion is the existence of a human resource policy?
   i. How, in your opinion, can the non-existence of a proper human resource policy affect you and the overall performance of the immunization program.

2. What is your opinion about the training/orientation session that you received after being hired?
   Prompts and probes:
   a. Was a comprehensive training provided?
   b. Do you think the training was sufficient or not?
   c. Was there anything that you think should have been a part of the training and it was not?

ANSWERABILITY

1. What are your views on the human resource development activities in the program?
   Prompts and probes:
   a. Do they exist and is a separate budget allocated for them?
   b. Do activities like trainings, refreshers, and performance planning and performance evaluation exist?
   c. Who is responsible for human resource development? Is there a designated department or person responsible for HRD
   d. Is it important to have human resource development activities and how, in your opinion, they can affect yours and the overall program performance?

2. What is the situation of employee turnover and what are the reasons for it?

3. Are you satisfied with the number and capacity of staff present at your level (position) for an effective implementation of immunization program?
   Prompts and probes:
   a. Can you easily cover the target area for delivering the immunization services?
   b. Is there a designated person responsible for ordering, keeping record and storing vaccines?
   c. Is there a designated person responsible for the receipt, Storage of vaccines and the recording of vaccines?

4. Are you satisfied with the material and monetary resources provided to you for successfully conducting your jobs?
   a. Ask specifically about office space, vaccine, storage box/refrigerators, syringes, information material, logistics and daily allowance

5. What is your opinion about the acceptance of the immunization program within your community and the cooperation from the community?
   Prompts and probes:
   a. Is community support and acceptance important for your and overall program performance?
   b. Is community supportive of the vaccination program?
   c. Are there any social mobilizers within the community?
   d. Are there any influential groups that make the working environment security compromised for you?
   e. Are there any groups of influential individuals in your community that do not support vaccination?

ENFORCEMENT

1. How satisfied are you with your salary and benefits structure?
   Prompts and probes:
   a. Is salary disbursed timely?
   b. Is salary enough to meet the daily living
   c. Is there a well-defined benefit system such as health insurance, paid leaves, vacations, regular raise, and promotions etc?

2. What is the existing procedure for supervision and assistance?
   Prompts and probes:
   a. Is supervisory plan in place for all levels?
   b. Is there a clear line of authority?
   c. Do supervisors recognise and practice their roles?
   d. Do supervisors take part in work planning and performance evaluation with their supervisees?
   e. Are you assisted by your supervisor in professional development and learning new skills?
   f. If supervisor is not available, what is your source of information (if no guidelines are available)?

3. What role can performance and workload-based incentives and appreciation for the staff, play in improving the motivation?
   Prompts and probes:
   a. Is performance evaluation done regularly?
   b. How often is your work acknowledged or appreciated?
   c. Do you get any performance-based rewards (monetary or non-monetary)?
   d. How often is the data that you submit analysed at the higher levels and proper actions taken for the problems?
   e. Is any feedback provided based on the data submitted and problems identified by you?

4. What is your opinion about the disciplinary measures taken by the higher officials in response to poor performance?
   Prompts and probes:
   a. Do and what disciplinary measure exist?
   b. Who takes the disciplinary actions if any?
   c. What is the severity of the disciplinary action if any?

GENERAL

1. What in your opinion can be the best measures to increase motivation and ultimately the outcome of the staff?
2. What in your view will be the five major challenges being faced by the immunization program in Baluchistan related to accountability and implementation of services?
3. What in your view, can be the best solutions to the problems you identified above? Please suggest solution to each of the above problems and also indicate the responsible entity

CLOSING
This concludes the interview. Thank you for your time and participation.
